# A Case of Self-Emasculation

**Published:** 1846-06

**Authors:** C. Gliddon Young

**Affiliations:** Greenwood, La.


*A Case of Self-Emasculation.* By C. Gliddon Young, M.D., of Greenwood, La.

I was called in the fall of 1837, to see Mr. P., a married man, in respectable circumstances, aged about 35 years, the father of five children, who, in a state of mental despondency, produced by religious excitement and domestic troubles, had emasculated himself. He was an exemplary member and class leader in the Methodist church, and was much in the habit of shouting, which was so disagreeable to his wife that she required him to desist, on pain of forfeiting his connubial rights. Shortly afterwards she discovered that he had deliberately sharpened his knife and maimed himself as above mentioned; taking out first one testicle and then the other, after the manner of castrating pigs. As he was ignorant of the danger of hemorrhage, or the means of guarding against it, he had cut the cord directly across, and both spermatic arteries were bleeding when I got to him. I found him almost exhausted from the loss of blood; he had fainted several times; the scrotum was filled with coagula. I quickly cleared the coagula from one side, and found that the cord had retracted so far within the abdominal ring as to be scarcely within reach; but a tenaculum introduced by the side of my finger enabled me to bring down and secure the bleeding arteries, which I did successively. By a little stimulus he was revived, and his recovery took place without a single untoward symptom.

May, 1846.

